# Ductal Carcinoma *In Situ*: Recent Advances and Future Prospects

**DOI:** 10.1155/2012/347385

**Published:** 2012-05-17

**Authors:** Kelly Lambert, Neill Patani, Kefah Mokbel

**Affiliations:** ^1^The Breast Unit, University Hospitals Leicester, Leicester LE3 9QP, UK; ^2^The London Breast Institute, The Princess Grace Hospital, London W1U 5NY, UK

## Abstract

*Introduction.* This article reviews current management strategies for DCIS in the context of recent randomised trials, including the role of sentinel lymph node biopsy (SLNB), adjuvant radiotherapy (RT) and endocrine treatment. *Methods.* Literature review facilitated by Medline, PubMed, Embase and Cochrane databases. *Results.* DCIS should be managed in the context of a multidisciplinary team. Local control depends upon clear surgical margins (at least 2 mm is generally acceptable). SLNB is not routine, but can be considered in patients undergoing mastectomy (Mx) with risk factors for occult invasion. RT following BCS significantly reduces local recurrence (LR), particularly in those at high-risk. There remains a lack of level-1 evidence supporting omission of adjuvant RT in selected low-risk cases. Large, multi-centric or recurrent lesions should be treated by Mx and immediate reconstruction should be discussed. Adjuvant hormonal treatment may reduce the risk of LR in selected cases with hormone sensitive disease. *Conclusion.* Further research is required to determine the role of new RT regimes and endocrine therapies. Biological profiling and molecular analysis represent an opportunity to improve our understanding of tumour biology in DCIS to rationalise treatment. Reliable identification of low-risk lesions could allow treatment to be less radical.

## 1. Introduction

### 1.1. Diagnosis

The introduction of national mammographic screening programmes and the increasing use of digital mammography and MRI have dramatically changed the clinical presentation of DCIS. Prior to this, DCIS made up a small proportion of all breast malignancy and was only diagnosed in patients presenting with a palpable mass, pathological nipple discharge, or occasionally found as an incidental biopsy finding [1, 2]. In contrast, it is now most frequently identified in asymptomatic women with screen-detected micro calcifications [3] and makes up a larger proportion of breast malignancy. Approximately one fifth of all screen-detected breast cancers are now DCIS [4].

Although the rates of all breast malignancy have increased with time, between 1980 and 1995, Western countries have experienced a four-fold “increase” in the incidence of DCIS specifically, particularly in women of screening age [5].

Data from a systematic review of 374 studies reported the pooled incidence of DCIS in the early 1970s as 5.8/100000 and this had risen to 32.5/100000 in 2004 [6]. A higher proportion of the cases post screening were non comedo DCIS, which is considered less aggressive.

Screening and cancer registry data from Norway including 2.3 million women reported in 2010 showed an increase in incidence of DCIS from 4/100000 before the introduction of screening to 11/100000 postintroduction. In women of screening age, the proportion of DCIS within breast malignancy rose from 5% to 13%. Age-standardised rates of all breast cancer including DCIS increased over time in those of screening age, but a large peak at the point screening was introduced, subsequent drop in incidence (but not to prescreening levels), then a steady climb over time. Rates were also higher in prevalent as opposed to incident screens [7].

These studies seem to suggest that the introduction of screening is largely responsible for the apparent increased incidence of DCIS in recent times, but that the stage of the disease may be much earlier and possibly less clinically relevant [6].

The trend is likely to continue with further technological advances, including the transition from analogue to full-field digital mammography (FFDM) and the development of computer-aided detection (CAD) [8].

Although the role of MRI in the management of DCIS is yet to be fully evaluated by randomised trials, it is being used to assess disease extent and distribution and to assess the contralateral breast [9, 10]. It is also used for early detection of invasive and noninvasive breast cancer in high-risk women [11]. A recent prospective observational study demonstrated MRI to be significantly more sensitive than mammography for the diagnosis of DCIS (92% versus 56%) [12].

MRI can overinterpret nonmalignant incidental lesions which may result in unnecessary interventions [13]. A recent retrospective report looking at MRI screening of high-risk women demonstrated an increased sensitivity of MRI for DCIS that was at least as good as the sensitivity for invasive disease [14]. This study examined two time periods (before 2001; 223 women and after 2001; 391 women) in one unit which used MRI and mammography to screen high-risk patients. After 2001, the unit acquired additional breast coils, better methods of data processing, and staff with appropriate specialist training. In the first period, 3.1% of screens were positive and 13% of these were DCIS. All were diagnosed by mammography. In the second period, 3.3% of screens were positive, 34% were DCIS. All of these were diagnosed by MRI and just one of these was also seen on mammography. The specificity of MRI was lower than mammography and significantly more patients were recalled for suspicious changes on MRI in the second study period than the first, but there was no significant difference in the numbers of biopsies performed.

**Figure 1:**
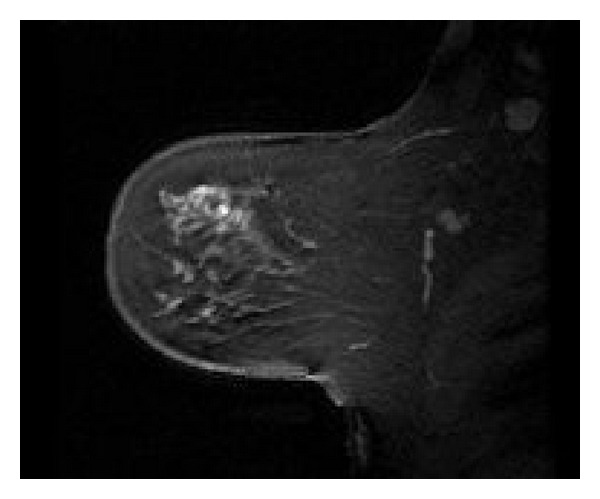
MRI appearance of recurrent DCIS.

Figure 1 shows regional ductal enhancement in the UOQ of the left breast anterior to a lumpectomy site in a 49-year-old female. This was recurrence picked up on screening MRI. Her mammogram was normal.

**Figure 2:**
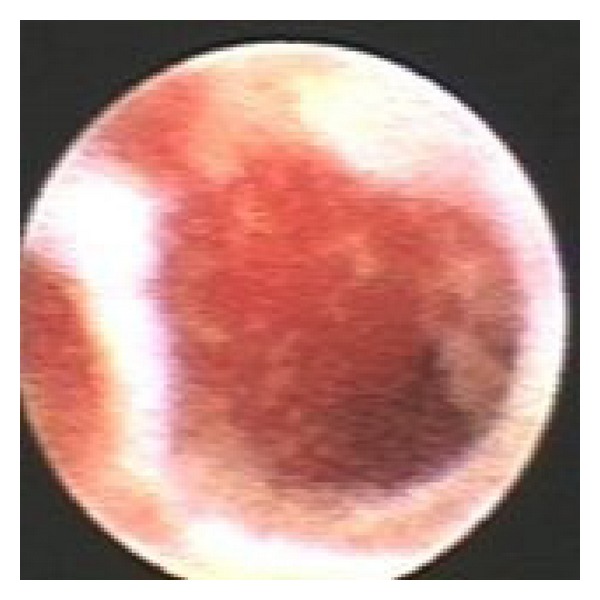
Appearances at mammary ductoscopy of DCIS.

Mammary ductoscopy has been used to directly visualise DCIS.  Figure 2 shows the appearance at mammary ductoscopy of histologically verified DCIS. However, this technique requires further investigation [15]. For example, it is limited by the fact that not all ducts are accessible from the nipple [16].

Currently, the preoperative diagnosis of impalpable lesions suspicious of DCIS requires either stereotactic or MRI guided core biopsy. Vacuum-assisted core biopsy (VACB) has been shown to increase the diagnostic yield and upgrade atypical ductal hyperplasia (ADH) to DCIS in approximately 25% of cases [17] and can be employed where standard core biopsy does not show DCIS yet the radiological changes are suspicious.

## 2. Pathological and Clinical Correlation

### 2.1. Classification

DCIS is defined by two features: firstly, the malignant epithelial proliferation is limited by the ductal basement membrane and secondly, stromal invasion is absent. DCIS behaves as a nonobligate precursor of invasive carcinoma and does not fully express the malignant phenotype [1]. The progression to invasive breast cancer is not completely understood and cannot be reliably predicted. Classification systems aim to reproducibly categorise lesions and to provide prognostic information to aid management decisions.

DCIS may be classified by grade, by architecture or morphology, by the level of differentiation, or by systems which use a combination of these factors [18].

Conventional histopathological types include comedo (tending to high grade cellular/nuclear features, often with central necrosis and calcification), solid, cribriform (with small holes or open spaces), and micropapillary (finger-like projections), however, lesions often demonstrate architectural and morphological heterogeneity [19, 20].

**Figure 3:**
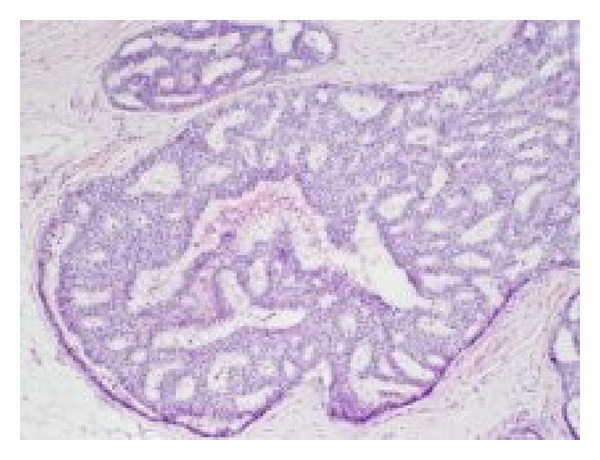
Low-grade DCIS.

**Figure 4:**
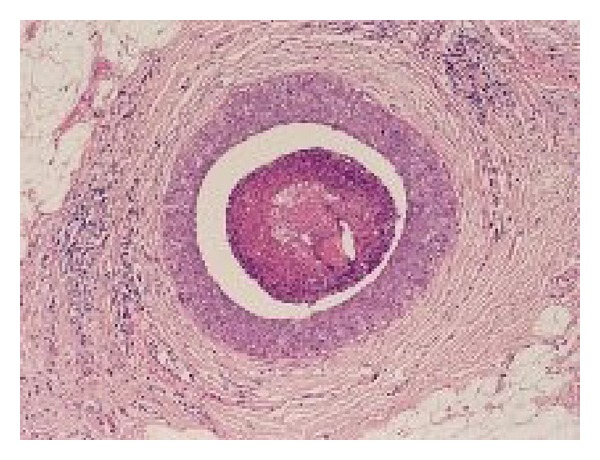
High-grade DCIS.

Cytonuclear grade is conventionally defined as low, intermediate, or high. It may vary between pathologists [21] and protocols have been developed to standardise reporting of grade [22]. Figures 3 and 4 show the characteristic features of low- and high-grade DCIS.

The “Comedo” subtype (high-grade, central confluent necrosis, and solid architectural pattern in >50% of the duct spaces) and the presence or absence of necrosis are important features and are incorporated into classifications such as the Van Nuys Index [23] and the Nottingham Grade [24].

All of the above classification methods as well as tumour size and the presence of absence of inflammatory changes have been found to be statistically associated with the risk of local recurrence in an independent pathological review of cases from the UKCCCR/ANZ DCIS trial [18]. This indicates their independent value in the classification of DCIS. Their combination and weight in terms of prognosis is yet to be defined.

### 2.2. Natural History

The elusive natural history of DCIS probably reflects the biodiversity of the condition. Preinvasive lesions do not invariably progress to invasive malignancy [25].

The natural history of small, noncomedo, low-grade DCIS treated by biopsy alone has been evaluated in long-term followup studies. After a median of thirty-one years, 39% of patients developed invasive breast cancer, all of which occurred in the index quadrant and 45% of these patients died of metastatic disease [26]. The overall progression to invasive breast cancer has been reported to range from 14% to 75% [27].

There is a wide body of evidence on the risk factors for breast cancer overall, but evidence on risk factors specific to DCIS is limited. However, it does appear that the same factors are involved as for invasive disease; high mammographic density, significant family history of breast-cancer, age, obesity, and high lifetime exposure to oestrogens [11].

Hence, it would seem that patients who receive no treatment beyond a diagnostic biopsy remain at significant risk of progression to invasive disease and that DCIS represents a precursor of invasive cancer. Increased risk has been demonstrated in lesions of all nuclear grade. On the other hand, a significant proportion of DCIS lesions do not progress. As diagnostic frequency continues to increase, there is an impetus to accurately identify clinically relevant lesions in order to rationalise management.

### 2.3. Clinical Characteristics

Women with palpable DCIS and those who present symptomatically exhibit higher rates of LR than mammographically detected cases [28, 29]. Some screen-detected lesions may, therefore, be less relevant than symptomatic lesions [30].

One study identified a family history of invasive breast cancer as a significant predictor of LR in women with DCIS treated with BCS and RT [31]. Previous therapy with oestrogens, either contraceptives or hormone replacement therapy, is also reported to be a significant predictor of LR [32].

Young age (<40 years) has emerged as an independent risk factor for LR after BCS with or without adjuvant RT [33]. LR has been reported to range from 11–31% in this group, with the lowest rates in mammographically detected lesions [33].

### 2.4. Pathological Characteristics

A meta-analysis of 44 trials has reported significantly increased pooled risk estimates for local recurrence after treatment for DCIS if the disease is classified as “comedo” type, multifocal, if the lesion is large or highgrade. Involved margins were associated with the highest increase in risk estimates and there was limited evidence that ER- and PR-positive HER2 negative disease is less likely to recur [34].

In the meta-analysis, the pooled risk estimates for size were derived from 7097 women. Lesions greater than 20 mm in size were compared with lesions less than 20 mm. The risk estimate for larger lesions was 1.63 (95% CI 1.30–2.06). Accurate and reliable measurement of DCIS can be challenging and several landmark studies have been criticised for their performance in this regard [29, 35, 36]. The large numbers in the meta-analysis will have helped to mitigate this factor. The same study reported the summary risk estimate for multifocal versus unifocal disease to be 1.95 (95% CI 1.59–2.40) from analysis of 3895 patients [34]. It is possible that the total area of DCIS in multifocal lesions is greater than unifocal disease and that the difference in local recurrence could be secondary to this.

Involved margins are associated with an increase in LR, in patients treated by BCS alone, and in those who also undergo RT [37–39]. Consensus has yet to be reached with regard to optimal margin width [36]. The presence of DCIS at the surgical margin is associated with the identification of residual DCIS in 40–82% of reexcision specimens, and is correlated with margin width: 41% at <1 mm, 31% at 1-2 mm and 0% with ≥2 mm of clearance [40]. The French National Guidelines recommend surgical margins of ≥3 mm, and reexcision for margins <1 mm [41]. A meta-analysis reporting the effect of margin status on local recurrence after BCS and RT concluded that a margin width of ≥2 mm was significantly superior to lesser margins (odds ratio (OR) = 0.53, 95% CI 0.26–0.96). However, there was no added value associated with clearance ≥2 mm compared to >5 mm (OR = 1.51, 95% CI 0.51–5.0) [42]. Despite this, total excision volume, independent of margin clearance, has also been correlated with LR. Following BCS for DCIS, the Joint Centre Experience reported LR rates at 5 years of 9% and 0% for volumes <60 cm^3^, and >60 cm^3^ respectively [43]. Excision volumes <60 cm^3^ have been shown to increase the relative risk of LR in women under 45 years [33]. Margins were associated with the largest difference in the risk estimates for local recurrence in the meta-analysis by Wang [34]. The summary risk estimate for women with involved margins was 2.25 (95% CI 1.77–2.86). 

High nuclear grade is associated with a greater risk of LR. In Wang's meta-analysis [34], 10,526 women were included in the analyses relating to grade, the summary risk estimate for high grade versus non-high-grade disease was 1.81 (95% CI 1.53–2.13).

The combination of nuclear grade and comedonecrosis is strongly associated with the risk of LR after BCS [23]. In the same meta-analysis of 9332 women with DCIS, the summary risk estimate of comedo-necrosis versus none for invasive breast-cancer recurrence was 1.71 (95% CI 1.36–2.16) [34].

A recent population-based case-control study found that comedo-type DCIS shares a similar profile of hormonal and reproductive risk factors to IBC, including ≥10 years of oral contraceptive intake and an inverse association with ≥3 full-term pregnancies. These findings were in contrast to those for noncomedo lesions, providing some further support for the differential management of DCIS lesions [44]. The significance of comedo-type as a risk factor for LR has resulted in its inclusion in prognostic indices [45, 46].

High-grade DCIS which is oestrogen receptor (ER) and progesterone receptor (PR) negative is significantly associated with HER2 and p53 positivity [47]. HER2 positivity and ER/PR negativity are individually associated with increased risk of LR [48]. HER2 overexpression represents an aggressive biological subtype of DCIS, correlating with high grade, p53 expression, and hormone receptor negativity. Hormone receptor positivity has been associated with low-grade DCIS. In a recent case series, HER2 was found to be superior to lesion size or nuclear grade in predicting concurrent invasive disease. DCIS that overexpressed HER2 was 6 times more likely to be associated with invasive disease (OR 6.4, *P* = 0.01) [49].

In the Wang meta-analysis [34], higher rates of local recurrence were seen in ER/PR negative, HER2, positive-patients but the differences were not statistically significant.

### 2.5. Molecular Characteristics

Various molecular markers have been studied in DCIS as possible predictive or prognostic factors for progression to invasion or for the development of invasive recurrences.

In invasive breast cancer, classifications based on biological profile (derived from gene profiling and correlated with immunohistochemical profile) rather than morphology have been developed and shown to correlate with prognosis. In order; Luminal A, Luminal B, Triple negative, and Basal Type invasive breast cancers are associated with a worsening prognosis [50]. The same profiles have been demonstrated in DCIS [51]. More work is now needed to establish whether these profiles influence the likelihood that an area of DCIS will progress.

Chromosome-wide comparative genomic hybridization has shown DCIS to be a genetically advanced lesion with alterations corresponding to adjacent invasive disease and independent pathways of genetic evolution [52]. A distinctive molecular portrait of each lesion can be obtained by gene expression profiling using complementary DNA micro-arrays [53].

One such study has identified a gene expression classifier of 35 genes which differ between DCIS and IBC and a further 43 genes distinguishing well-from poorly differentiated DCIS [54]. Protein expression profiling can similarly be undertaken using matrix-assisted laser desorption/ionization (MALDI) or surface-enhanced laser desorption/ionization (SELDI). Although the relevance of each parameter may not be fully understood, combinations of features may enable the biological profiling of DCIS lesions into groups of similar natural history and prognosis.

Balleine et al. recently reported on a binary molecular grading scheme for DCIS, based on expression at 173 oligo-nucleotide probes. Two conventional parameters amenable to routine evaluation (nuclear grade and Ki67 score) were capable of accurately assigning lesions into low or high molecular grade [55].

Proteomics analysis of DCIS and normal breast tissue has also identified differential expression patterns, distinct from previous nucleic-acid-based studies [56]. Expression of Syndecan-1, E-cadherin, and c-met have recently been shown to be associated with angiogenic and lymphangiogenic factors in DCIS, including endothelin A and B receptors, vascular endothelial growth factor (VEGF)-A/C, and fibroblast growth factor receptor (FGFR)-1 [57]. In addition to their potential use for prognostication, putative molecular targets may enable directed therapy in the future.

Intuitively, molecules such as matrix-metalloproteinases (MMPs) and tissue inhibitors of matrix-metalloproteinases (TIMPs) that influence the invasion of stroma and basement membrane should be important in the progression of DCIS to invasive breast cancer. Significantly different expression profiles of MMPs and TIMPs have been noted in DCIS, admixed DCIS, and invasive breast cancer [58]. More work is needed to understand the role these molecules have in progression to invasive cancer but their expression profiles could help determine lesions that should be treated more aggressively.

## 3. Management

It is possible that not all DCIS needs to be treated aggressively as not all DCIS will become invasive. In particular, small, low-grade lesions detected by screening may fit into this group. Management strategies need to consider the breast and axilla, the need for adjuvant RT, and the role of systemic adjuvant therapy. Treatment of the breast can involve BCS (with or without RT) or mastectomy (Mx). Axillary surgery, even SLNB, warrants particular caution in view of their low yield and potential for harm. Adjuvant systemic treatments have mainly involved oestrogen blockade with Tamoxifen. The optimal management of DCIS remains controversial [59].

### 3.1. Surgery

Complete excision of DCIS with clear margins is the most important factor in reducing the risk of LR. Mx is indicated for large tumours (>4 cm depending on breast size), multicentric lesions, inadequate margins after BCS, local recurrence after BCS (particularly with prior RT), and patient preference. Mx affords excellent local control, approximately 98% at 7 years, with an overall recurrence rate of 1.5% [60].

In England and Wales between 1990 and 2001, the absolute number of mastectomies for *in situ* disease increased by 400%, corresponding to the introduction of national screening [61]. The relative rate of Mx for DCIS has been decreasing over the last three decades and the procedure is now undertaken in approximately one third of patients [62]. The French Survey reported Mx rates of 10% for lesions <10 mm compared to 72% for >20 mm, and 11% for low-grade compared to 54% for high-grade lesions. The authors justify an Mx rate of 50% for patients <40 years by the lifetime risk of LR in those undergoing BCS despite adjuvant RT [62].

If patients do require Mx for DCIS, an immediate breast reconstruction is relatively uncomplicated as postmastectomy radiotherapy and lymph node dissection will not be required [63].

BCS combined with RT is an acceptable treatment option for smaller, unifocal areas of DCIS. There is probably not enough evidence to justify BCS without RT routinely. Significant numbers of patients undergoing BCS alone develop LR, of which approximately half are invasive and up to one fifth ultimately metastatic. The literature reveals an overall LR rate of approximately 28% at 7 years, around 45% of which are invasive [37–39, 64–68]. There is also evidence that mammographically detected DCIS treated by BCS alone has unacceptable rates of LR (10-year LR rates were 27.8%, 22%, and 19%, resp., of which approximately 35% were invasive) [68–70].

### 3.2. Radiotherapy

The benefit of adjuvant RT, in terms of reduced LR in those undergoing BCS, has been demonstrated by several large randomized controlled trials. However, clear margins are necessary even if RT is given to obtain acceptable rates of LR [37, 38, 71, 72]. There remains a lack of level-1 evidence supporting the omission of adjuvant RT in selected low-risk cases.

The National Surgical Breast and Bowel Project (NSABP B-17) trail randomized 818 patients after BCS surgery for DCIS, to either whole breast RT or no further treatment [35]. After a median followup of 129-months, of the 403 women treated by wide local excision alone, there were 124 local recurrences (31.7%), 67 of which were invasive (54%). Of the 410 women treated by wide local excision and RT, 61 local recurrences were observed (15.7%) of which 29 were invasive (48%, *P* = 0.001). Despite the fact that RT was associated with a 57% reduction in LR (both invasive and *in situ*), no differences were observed in the rates of distant recurrence and overall survival.

An analysis of long-term data from the NSABP B-17 and NSABP B-24 trials [73] showed that at 15 years the radiotherapy treated patients still had significantly fewer local recurrences and this effect had increased over time. Of those that did recur, 54% were invasive, and for these patients, overall survival was lower (HR of death = 1.75, 95% CI = 1.45 to 2.96, *P* < 0.001).

The European Organisation for Research and Treatment of Cancer (EORTC) conducted a similar study recruiting 1010 patients [29]. After a median followup of 126 months, local relapse-free rates were 85% in the RT group and 74% in the control group (HR: 0.53, *P* < 0.0001). *In situ* LR rates were 7% and 13%, respectively, and invasive LR rates were 8% and 13% respectively [74]. Consistent with the NSABP B-17 trial findings, the absolute reduction of LR by RT increased with time from 7% at 4 years to 11% at 10.5 years. In univariate analysis, RT showed a statistically significant benefit in all subgroups of patients, but the size of this benefit varied. The authors observed a 23.5% and 42.7% LR rate for complete and incomplete/doubtful excisions, respectively, in the lumpectomy alone group, versus 14.7% and 24.7% for patients receiving adjuvant RT. Indicating the importance of clear margins even with RT.

The UK/ANZ DCIS trial involved 1701 patients treated by BCS, with subsequent randomisation to RT and/or Tamoxifen [75]. There were four treatment groups: BCS alone, BCS + RT, BCS + TAM, and BCS +  RT +  TAM. 90% of the participants were 50 years or older with screen detected DCIS. After a median followup of 53 months, the respective rates of LR were 22%, 8%, 18%, and 6%. Adjuvant RT was associated with a significant reduction (hazard ratio (HR) = 0.38, *P* < 0.0001) in all ipsilateral tumour recurrence (invasive or DCIS). RT reduced the risk of DCIS by 64% (*P* = 0.0004) and invasive cancer by 55% (*P* = 0.01). Long-term followup data has since been reported for this trial [76]. At a median followup of 12.7 years, the treatment effects are similar in magnitude.

The Cochrane Collaboration has recently published a systematic review of four adjuvant RT trials: NSABP 2001 [35], EORTC 2006 [29], UK/ANZ DCIS 2003 [75] and the Swedish DCIS 2008 [77]. With regard to LR, they report a 51% pooled risk reduction for DCIS (HR 0.61, 95% CI 0.39–0.95, *P* = 0.03) or invasive cancer (HR 0.50, 95% CI 0.32–0.76, *P* = 0.001). After a median followup ranging from 4.4–10.5 years, the LR rate for those receiving RT was 11.6% compared to 23.9% for BCS alone, resulting in a number needed to treat (NNT) of 9 patients to prevent one LR. Although there was no attributable increase in mortality, long-term RT complications were poorly reported by the trialists [78].

A further meta-analysis also concluded that adjuvant RT significantly reduces the risk of LR after BCS—by approximately 60%, with most benefit to patients with high-grade lesions and positive margins. RT did not significantly alter the rate of distant metastases or overall survival [79].

Overall, LR rates have been reported to range from 2.7% to 18.9%, averaging 10% at 7 years, with invasive LR accounting for approximately 60% [80]. However, the methodological quality of several trials has been criticised, particularly in terms of the treatment of unclear margins, the methodology and design of the studies, and the validity of conducting posthoc secondary retrospective analyses [29, 35]. Whilst some of these issues can be resolved by meta-analysis, others are being addressed by current studies.

Although it has been long been proven that radiotherapy after mastectomy for invasive breast-cancer reduces local recurrence [81], good evidence that this in turn leads to reduced mortality took much longer to be published [82, 83]. DCIS is associated with a better prognosis than invasive breast cancer and, therefore, proof of a survival benefit with radiotherapy may take time to establish.

Strategies such as a boost of RT to the tumour bed are used in IBC. There is no evidence that this reduces LR in DCIS. A study of 75 patients treated by BCS+RT, including 20 women receiving an additional 10 Gy boost to the tumour bed, identified no improvement in LR reduction after a median followup of 81 months [84]. The efficacy of other novel strategies including partial breast RT in the context of DCIS has yet to be evaluated [71, 72, 85]. Accelerated partial breast irradiation (APBI) aims to provide comparable local control to whole-breast RT with reduced morbidity. In the largest study group of patients with DCIS (*n* = 194) treated with the MammoSite device, the 3-year actuarial LR rate was 0% in the first 48 cases enrolled compared to 2.04% in IBC (*n* = 352); median followup 37.5 months [86]. Another recent study of 126 DCIS cases evaluated balloon-based brachy therapy, with either MammoSite or Contura catheter. After a median followup of 40 months, the LR rate for the first 50 consecutive cases was 0.02% with a 3-year actuarial rate of 2.15% [87].

The ECOG group (Eastern Cooperative Oncology Group) prospectively studied 565 nonrandomised patients with single areas of DCIS less than 2.5 cm in size treated by breast conserving surgery alone, with margins of greater than 3 mm and split these patients into a low and intermediate group versus a high-grade group [88] attempt to determine a sub-group of patients in whom RT could be omitted. After a median followup of 6.7 years, the local recurrence rate was 6.1% (95% CI: 4.1–8.2%) in the low to intermediate grade group and 15.3% (95% CI: 8.2–22.5%) in the high grade group. On the basis of this, the authors suggest that small, low-grade lesions excised with generous margins by breast-conserving surgery may not need radiotherapy. The authors did caution that longer followup and additional study would be needed to confirm this and raise the point that recurrences from low-grade lesions may present later.

The Radiation Therapy Oncology Group trial (98-04) was a randomised trial designed to assess the need for radiotherapy for DCIS in patients with “low-risk” but unfortunately closed due to nonaccrual. A recent study attempted to account for the nonrandomisation in the ECOG DCIS study by comparing two groups of patients (low and intermediate or high grade DCIS) that were treated with breast-conserving surgery and radiotherapy with the two groups in the ECOG study [89]. In these 263 patients, with similar length of followup, there was a reduction of more than 70% in the local recurrence rates with radiotherapy.

More evidence is needed to confirm if there is a subgroup of patients with DCIS that do not need radiotherapy after breast conservation.

### 3.3. Endocrine Therapy

Hormonal therapies (mainly Tamoxifen) are the main stay of systemic adjuvant therapy in DCIS.

The NSABP B-24 trial was designed to assess the benefit of Tamoxifen for 5 years versus placebo after BCS and RT for DCIS [90]. After 7 year median followup, the LR rates were 11.1% and 8% in the placebo and Tamoxifen groups, respectively (*P* = 0.02). The absolute reduction was significant for invasive LR. There was a significant excess of endometrial cancer and thromboembolic events in the Tamoxifen group. No significant benefit was observed in the following groups: age >50 years, *in situ* LR, complete local excision, and absence of necrosis. The overall mortality was not affected [91]. A posthoc analysis of ER status demonstrated that efficacy was limited to the 77% of cases which were ER positive [92].

The UK/ANZ DCIS trial also assessed the effect of adjuvant treatment with Tamoxifen after BCS and RT for DCIS. The results were originally reported after a median followup of 4.4 years [75]. At this point, there was no significant difference in the incidence of invasive breast cancer events in the Tamoxifen-treated patients. However, the total number of DCIS events (ipsilateral and contralateral) was significantly reduced by Tamoxifen (6% versus 10%, *P* = 0.03). After a median of 12.7 years followup [76], a significant difference in all new breast events in Tamoxifen treated patients was seen (HR 0.71, 95% CI 0.58–0.88;

*P* = 0.002). Tamoxifen reduced both recurrent ipsilateral DCIS ( HR 0.70, 95% CI 0.51–0.86; *P* = 0.03) and contralateral tumours (HR 0.44, 95% CI 0.25–0.77; *P* = 0.005). No significant reduction in ipsilateral invasive disease has been proven (HR 0.95, 95% CI 0.66–1.38; *P* = 0.8).

There is, therefore, good data that Tamoxifen reduces local recurrence and the risk of contra-lateral tumours in DCIS treated by BCS and RT. Some DCIS is probably low-risk enough to omit it, but clear evidence on this is lacking.

There is currently only limited data on the use of aromatase inhibitors in DCIS.

Trials are ongoing to determine if Aromatase inhibitors are superior to Tamoxifen in the adjuvant setting after breast conserving surgery for DCIS (NSABP B-35 and IBIS II).

Recently, inhibition of cyclo-oxygenase 2 (COX-2), implicated in epithelial-stromal interactions and promoting the progression of DCIS, has been evaluated using nonsteroidal anti-inflammatory drugs (NSAIDS). Results from experimental studies were encouraging [93, 94] but were not supported by the ERISAC trial [95].

### 3.4. Sentinel Lymph Node Biopsy

Pure DCIS does not exhibit lymphatic or vascular invasion so surgical staging of the axilla is not necessary [59]. However, lesions thought to be noninvasive on core biopsy are upgraded in 10–33% of cases on final postoperative histology [96, 97] and lymph node positivity has been reported in 1-2% of patients (20) (which may be attributable to “missed” invasive foci) [27]. A relative indication for SLNB in DCIS is patients undergoing Mx, as these patients are likely to have risk factors for occult invasion—large or multifocal lesions, high-grade, mastectomy for recurrent disease [97].

Retrospective analyses from the NSABP B-17 and B-24 trials support the strategy of avoiding routine axillary surgery in DCIS due to low yield and risk of morbidity [98, 99].

### 3.5. Local Recurrence

If LR occurs after DCIS, it may be *in situ* or invasive. It occurs at the site of the original lesion or within the index quadrant in 75–80%. The risk decreases as the extent of primary treatment increases (BCS, BCS + RT, Mx). Ironically, LR can be more aggressive in those who were treated more aggressively. Whereas 40–50% of LR is invasive after BCS, LR is almost always invasive following Mx. This may reflect the fact that recurrence after BCS often presents as an incidental finding of *in situ* disease during surveillance mammography, whereas postMx ipsilateral mammographic screening is obviously not undertaken and recurrence is likely to present at a more advanced stage and relies on clinical detection [100]. The prognostic implications of invasive LR are significantly worse than *in situ* recurrence. In particular, the overall risk of metastasis has been reported to be 0–3.6% for *in situ* LR, compared to 13.2–18% after invasive LR [37, 38, 71, 72, 101]. The rate of axillary lymph node involvement with invasive LR ranges from 11–30% [37, 38, 71, 72]. 

Completion Mx is indicated following LR within the breast when reexcision would be cosmetically unacceptable, or when LR is confirmed to be invasive and for those with an absolute or relative contraindication to RT (i.e., previous adjuvant RT). In the NSABP B-17 trial, the Mx rate for LR was 48% in the BCS group and 62% in the BCS + RT group [35], consistent with similar studies reporting rates of 52.8% and 74.7%, respectively, [37, 38]. Overall, salvage Mx rates range from 64–84% [37, 38, 71, 72].

### 3.6. Special Clinical Scenarios

Management of the elderly DCIS patient (particularly those over 70 years) is not strongly evidence based as this group has often been excluded from important trials and screening programs [35, 45, 46, 71, 72, 90, 91].

Women exposed to thoracic radiation, including prior treatment for haematological malignancies, are at risk of developing secondary tumours, with breast cancer representing the most common solid lesion and DCIS accounting for 11–17.7%. The risk is significantly increased at adolescence and young adulthood with a median onset interval of 16 years. In one study, the majority of these patients were treated with Mx, however, 29% underwent BCS ± RT [102]. 

Male DCIS has been reported in approximately 300 cases, however, the incidence of DCIS within IBC ranges from 0% to 17% with an average of 7% [103, 104]. Patients may present with a subareolar mass, Paget's disease, or serosanguineous nipple discharge. Optimal control is achieved with simple mastectomy and lumpectomy alone has been associated with a higher rate of LR.

### 3.7. Future Strategies

Minimally invasive interventions for breast cancer seek to redress the balance between benefit and risk and may, therefore, be of particular use in asymptomatic patients with low-risk lesions or patients deemed unfit for conventional management. Image-guided radiofrequency ablation therapy (RFA) has been demonstrated in pilot studies to be effective with few complications and a favourable safety profile. However, complete ablation may not achievable in all patients and exhaustive histological specimen analysis is not possible. Furthermore, current imaging modalities are relatively imprecise at delineating the extent of DCIS and predicting/confirming complete ablation [105].

## 4. Summary

DCIS should be managed within the multidisciplinary team and management tailored to patient and tumour factors. Local control depends upon adequate surgical clearance, and in order to reduce the risk of LR, surgical margins of at least 2 mm should be achieved. SLNB can be considered in patients with a high-risk of occult invasive disease. RT following BCS significantly reduces LR, particularly in those at high-risk. There remains a lack of level-1 evidence supporting the omission of adjuvant RT in selected low-risk cases. Large, multicentric, or recurrent lesions (particularly in cases of prior RT) should be treated by Mx and immediate reconstruction should be discussed. Adjuvant Tamoxifen may reduce the risk of LR in patients with hormone sensitive disease. Further research is required to determine the role of contemporary RT regimes and endocrine therapies. Biological profiling and molecular analysis represent an opportunity to improve our understanding of the tumour biology of this condition and rationalise its treatment. Reliable identification of low-risk lesions could allow treatment to be less radical or safely omitted.

## 5. Search Strategy and Selection Criteria

Articles were identified by searches of Medline, PubMed, Embase, and Cochrane databases up to September 2011 using the terms: “DCIS” or “ductal carcinoma *in situ*” and “treatment” or “management” or “surgery” or “radiotherapy" or “radiation” or “mastectomy” or “sentinel lymph node biopsy” or “natural history” or “Tamoxifen” or “recurrence” or “invasive.” Studies identified were screened for those that focused on DCIS treatment. All randomized controlled trials and large retrospective series were included. The references in this review were selected to provide a balanced and representative overview of a complex subject with an extensive base of published work.
